# Dynamic Causal Modeling Self-Connectivity Findings in the Functional Magnetic Resonance Imaging Neuropsychiatric Literature

**DOI:** 10.3389/fnins.2021.636273

**Published:** 2021-08-11

**Authors:** Andrew D. Snyder, Liangsuo Ma, Joel L. Steinberg, Kyle Woisard, Frederick G. Moeller

**Affiliations:** ^1^Institute for Drug and Alcohol Studies, Virginia Commonwealth University School of Medicine, Richmond, VA, United States; ^2^Department of Psychiatry, Virginia Commonwealth University School of Medicine, Richmond, VA, United States; ^3^Department of Radiology, Virginia Commonwealth University School of Medicine, Richmond, VA, United States; ^4^Virginia Commonwealth University School of Medicine, Richmond, VA, United States; ^5^Department of Pharmacology and Toxicology, Virginia Commonwealth University School of Medicine, Richmond, VA, United States; ^6^Department of Neurology, Virginia Commonwealth University School of Medicine, Richmond, VA, United States

**Keywords:** dynamic causal modeling, intrinsic connectivity, extrinsic connectivity, effective connectivity, inhibitory interneuron, self-connectivity

## Abstract

Dynamic causal modeling (DCM) is a method for analyzing functional magnetic resonance imaging (fMRI) and other functional neuroimaging data that provides information about directionality of connectivity between brain regions. A review of the neuropsychiatric fMRI DCM literature suggests that there may be a historical trend to under-report self-connectivity (within brain regions) compared to between brain region connectivity findings. These findings are an integral part of the neurologic model represented by DCM and serve an important neurobiological function in regulating excitatory and inhibitory activity between regions. We reviewed the literature on the topic as well as the past 13 years of available neuropsychiatric DCM literature to find an increasing (but still, perhaps, and inadequate) trend in reporting these results. The focus of this review is fMRI as the majority of published DCM studies utilized fMRI and the interpretation of the self-connectivity findings may vary across imaging methodologies. About 25% of articles published between 2007 and 2019 made any mention of self-connectivity findings. We recommend increased attention toward the inclusion and interpretation of self-connectivity findings in DCM analyses in the neuropsychiatric literature, particularly in forthcoming effective connectivity studies of substance use disorders.

## Introduction

Dynamic causal modeling (DCM) is one means of computing and modeling neuronal effective (directional) connectivity between regions in the brain and likely the most commonly applied method to measure effective connectivity (Friston et al., 2019). DCM can also be used to measure self-connectivity which is also described in the literature as *intrinsic connectivity or endogenous connectivity*, technically described as intra-regional connections within any given volume of interest Friston et al. (2003). Despite increases in the relative rate of reporting these results in recent years, self-connectivity findings are much less commonly reported than extrinsic brain connectivity findings in the neuropsychiatric DCM literature.

Zeidman et al. (2019) stated that these findings are reported as the diagonal in the matrices of endogenous and modulatory effects defined in any given DCM – that is, every DCM will include these results. Self-connections provide only inhibitory (negative) influence on each region included in the model. Self-connections regulate the excitatory and inhibitory extrinsic (between-region) connections that may be estimated by the model and ultimately preclude the possibility of a runaway positive feedback loop in the neurologic model estimated by DCM. Extrinsic connections may contribute to the inhibitory regulation of any given region but, ultimately, it is the inhibitory (negative) inputs from the self-connections that control the gain of the regional response to extrinsic connection. Self-connections neurobiologically control the excitatory-inhibitory balance – in this sense, they are physically, chemically, as well as neuro- and electro-physiologically necessary for describing a viable model. For example, as the magnitude of the self-connection parameter increases, the net effect of extrinsic inputs on that region decreases and vice versa. Focusing on self-connectivity (intrinsic connectivity) in functional magnetic resonance imaging (fMRI) analyses, Friston et al. (2003) describe this effect as “augmenting or attenuating” the decay of synaptic activity.

At this time, most of the pre-existing discussion on the topic of self-connectivity pertains primarily to fMRI analyses. However, there is some discussion on utilizing self-connectivity findings in other imaging modalities, as well, namely electroencephalography (EEG) and magnetoencephalography (MEG). Brown and Friston (2012) discuss the modeling of self-connectivity using EEG data to model the self-inhibition of regions by superficial pyramidal neurons. They make brief reference of utilizing MEG and PET data to model similar interactions. We acknowledge the importance of these other neuroimaging modalities in the neuropsychiatric literature. But, our aim in this mini-review is to take account of the rate of reporting these kinds of results in the fMRI literature on neuropsychiatric disorders to understand better the trends in this aspect of DCM analyses.

## Methods

We conducted a literature review to identify references including dynamic causal analyses of fMRI data in the neuropsychiatric and neuro-behavioral literature. We further evaluated this subset of references for mention and consideration of self-connectivity findings, to include any mention, actual discussion in the text, and any kind of graphical representation of self-connectivity findings. We also conducted a more involved assessment of each reference recording more details on the number of participants, number of models, regions of interest (ROI), as well as number and type of connections reported to assess whether there may be any difference between the studies that reported self-connectivity findings and those that did not. We computed descriptive statistics of trends in reporting self-connectivity findings in the literature over the period of time considered. We also tabulated the number and type of imaging modalities utilized in this subset of the literature.

### Eligibility Criteria

We considered studies with: (1) analysis of fMRI data findings; (2) use of DCM to analyze imaging data; (3) variable group diagnosed with a neuropsychiatric disorder; and (4) publication in a PubMed-indexed journal. According to the protocol, neuropsychiatric disorders considered in the search included: attention deficit-hyperactivity disorder, anorexia, autism spectrum disorder, bipolar disorder, cannabis use disorder, cocaine use disorder, dementia, depression, gaming/gambling disorder, intellectual disability, obsessive-compulsive disorder, opioid use disorder, Parkinson’s disease, post-traumatic stress disorder, seizure disorder, schizophrenia, and tobacco use disorder.

### Information Search Strategy

The search was conducted by using search terms to identify articles that incorporated DCM analyses conducted on populations with neuropsychiatric disorders. To screen for pertinent articles, we used PubMed/MEDLINE for either “DCM” OR “DCM” AND one of the above neuropsychiatric disorders or pertaining acronym, to include the terms: “attention deficit-hyperactivity disorder,” “ADHD,” “anorexia,” “autism spectrum disorder,” “autism,” “bipolar disorder,” “cannabis use disorder,” “cocaine use disorder,” “dementia,” “depression,” “gambling disorder,” “intellectual disability,” “obsessive-compulsive disorder,” “opioid use disorder,” “Parkinson’s disease,” “post-traumatic stress disorder,” “PTSD,” “seizure disorder,” “schizophrenia,” OR “tobacco use disorder.” In essence, the goal was to identify articles including effective connectivity models pertaining only to a manageably broad interpretation of the neuropsychiatric literature. We also searched Google Scholar for additional references and included them regardless of PubMed indexing status to maximize the number of references captured for the review. The date of the last search was June 1st, 2020.

### Data Management

Literature search results were recorded from a Google Chrome internet browser using the Zotero reference manager application plug-in. Specific pertinent details for each reference were extracted and maintained in a Google Spreadsheet document. One author conducted the search and maintained the database of results. All authors discussed and reviewed the eligibility criteria and confirmed the viability of the references. Any duplicates from PubMed and Google Scholar were excluded upon review of the whole spreadsheet. There were no discrepancies or ambiguous inclusions in the reference list. All references identified were written in English.

### Data Extraction

All authors discussed and agreed upon the data to be extracted from the literature search. One researcher conducted the review and extraction of the pertinent data from the references obtained from literature search. Among the information collected were the following: neuropsychiatric disorder, report of self-connectivity results (binary), inclusion of discussion of self-connectivity results (binary), depiction of graphical representation of self-connectivity results (binary), number of subjects, number of controls, number of models reported, number of ROI, names of ROIs, number of extrinsic connections, number of self-connections, version of SPM used, version of DCM module used, type of model (deterministic, stochastic), and other information (authors, year of publication, and title). Report of, discussion of, and depiction of graphical representation of self-connectivity results were recorded using a binary variable (0 = not present, 1 = present). Numbers of subjects, controls, models reported, ROIs, extrinsic connections, and self-connections were all recorded as continuous variables. Neuropsychiatric disorders, names of ROIs, type of model were recorded with the appropriate designations.

### Data Analysis

We conducted basic descriptive statistics on the number of references including self-connectivity findings as well as the percent of self-connectivity findings overall as well as within each neuropsychiatric disorder included in the search. We also conducted unpaired *t*-tests of the continuous variables collected in an attempt to assess any potential differences between studies reporting self-connectivity findings and those without. Statistical tests were not conducted for number of self-connections given that the group not reporting these findings would have a count of zero by default.

## Results

The literature search revealed 81 references published during the years 2007 to 2019, spanning most of the neuropsychiatric conditions included in the aforementioned list. The distribution of neuropsychiatric disorders represented in the literature references are summarized in Table 1.

**TABLE 1:** Summary of neuropsychiatric disorders represented in the literature review by numerical count and percentage of total number of references.

#	Neuropsychiatric disorder	# Ref	# SC	% SC	Numbered reference list
1	Attention deficit-hyperactivity disorder	2	0	0%	Nagel et al., 2011; Posner et al., 2011
2	Anorexia	1	1	100%	Cha et al., 2016b
3	Anxiety	4	1	25%	Sladky et al., 2015; Cha et al., 2016a; Minkova et al., 2017; Neufang et al., 2019
4	Autism	5	1	20%	Sato et al., 2012, 2019; Gu et al., 2015; Prat et al., 2016; Stickel et al., 2019
5	Bipolar disorder	7	0	0%	Almeida, J. R. C., et al., 2009; Almeida, J. R., et al., 2009; Wu et al., 2014; Radaelli et al., 2015; Vai et al., 2015b; Dima et al., 2016; Zhang et al., 2018
6	Cannabis use disorder	2	0	0%	Ma et al., 2018a, 2019b
7	Cocaine use disorder	2	0	0%	Ma et al., 2014, 2018b
8	Dementia	3	0	0%	Sonty et al., 2007; Neufang et al., 2011; Rytsar et al., 2011
9	Depression	12	5	41.7%	Schlösser et al., 2008; Almeida et al., 2011; Desseilles et al., 2011; Goulden et al., 2012; Hyett et al., 2015; Musgrove et al., 2015; Posner et al., 2016; Vai et al., 2016; Li L. et al., 2017; Geng et al., 2018; Kandilarova et al., 2018; Zheng et al., 2018
10	Gambling/Gaming disorder	1	1	100%	Parkes et al., 2019
11	Obsessive-compulsive disorder	2	0	0%	van Velzen et al., 2015; Han et al., 2016
12	Opioid use disorder	1	0	0%	Ma et al., 2019a
13	Parkinson’s disease	6	1	16.7%	Husárová et al., 2013; Trujillo et al., 2015; Nackaerts et al., 2018a,b,c; Jastrzȩbowska et al., 2019
14	Post-traumatic stress disorder	3	1	33%	Nicholson et al., 2017a,b; Weng et al., 2019
15	Schizophrenia	25	7	28%	Mechelli et al., 2007; Crossley et al., 2009; Bányai et al., 2011; Deserno et al., 2012; Diwadkar et al., 2012, 2014; Chen et al., 2013; Curcic-Blake et al., 2013; Wagner et al., 2013, 2015; Fogelson et al., 2014; Bastos-Leite et al., 2015; Cui et al., 2015; Vai et al., 2015a; Dima et al., 2016; Lefebvre et al., 2016; Chahine et al., 2017; Dauvermann et al., 2017; Graña et al., 2017; Li B. et al., 2017; Nielsen et al., 2017; Zhang et al., 2017; Fang et al., 2018; Quarto et al., 2018; Zhou et al., 2018
16	Seizure disorder	5	2	40%	Vaudano et al., 2012, 2013; Klamer et al., 2018; Cook et al., 2019; Warren et al., 2019
	TOTAL	81	20	24.6%	

*SC, self-connectivity; # SC, number of articles, total, that have any mention of self-connectivity findings;% SC, percentage of articles within the disorder considered that have mention of self-connectivity findings.*

A broad range of neuropsychiatric and neurobehavioral disorders are covered in the DCM literature reviewed including mood, psychotic, addiction, and even eating disorders; some disorders are better represented with more numerous references than others. Schizophrenia, for example, was by far the most-studied among neuropsychiatric disorders considered using DCM of neuroimaging data with nearly a third of the references (26 out of 81, 32%). Depression was the second most prevalent with 12 (14.8%), followed by bipolar disorder (7, 8.6%) and Parkinson’s disease (6, 7.4%). The other disorders listed in the analysis include only a few to several references each and appear to be relatively less fully examined from an effective connectivity standpoint.

Of the total 81 references, 73 (90%) were found to have a graphical representation of a DCM model. Within the total 81 references, 21 references (25.9%) were found to have any kind of mention of self-connectivity findings – 19 (23.5%) of which included actual discussion of such findings and 14 (17.3%) had a graphical representation of self-connectivity findings.

**FIGURE 1:**
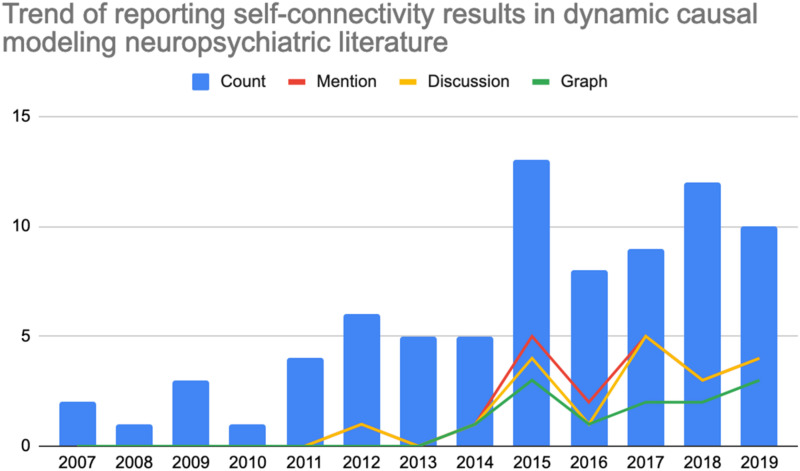
Count of references (left axis) by year within the literature review which (0, blue) total number of neuropsychiatric references reporting dynamic causal modeling findings per year; (1, red) count of references making any mention of self-connectivity results; (2, yellow) count of references including self-connectivity with the discussion; or (3, green) count of references demonstrating self-connectivity findings in graphical form.

Graphical depiction of the reference count by year as well as percentage mention of self-connectivity are included below in Figure 1. Note that the incidence is expressed as a percentage of references with mention, discussion, or a graphical representation for the particular year as a fraction of the total 81 references. For example, four references with mention of self-connectivity findings in 2019 would represent 4.9% of the total references reviewed reporting self-connectivity findings during that year alone.

There were no statistically significant differences between the studies reporting self-connectivity findings and those that did not for the other variables considered (e.g., number of subjects, number of ROI, number of models reported, etc.). There was, however, a general trend for later SPM versions for studies reporting self-connectivity findings.

## Discussion

The rate of reporting self-connectivity results in the DCM literature pertaining to neuropsychiatric disorders does not appear to have kept up with the relatively rapid adoption of DCM as an analytic technique in this area. Despite rapid adoption of DCM as an analytic technique in the neuropsychiatric literature – up from two publications in 2007 to 10 in 2019 – only 21 (25.9%) of the articles during this 13 year period have included any mention of self-connectivity findings. Under-reporting appears to have occurred in the context of what appears to be sufficient representation of the phenomenon of self-connectivity in the technical DCM literature. Because all DCM analyses include self-connectivity findings, some kind of statement should be included that summarizes these results, even non-significant ones. Regardless of statistical significance, these findings attribute important characteristics to the neurologic system being modeled using DCM.

Though the better-studied disorders feature self-connectivity reporting approaching 30–40% (e.g., depression, schizophrenia), none of them exceed even this modest threshold consistently. Perhaps most notable in this regard is bipolar disorder with 7 references identified articles reporting DCM results, zero of which reported self-connectivity findings. There are a few disorders with higher rates of reporting self-connectivity results (e.g., anorexia, gaming/gambling disorder), but these are also typically less well-examined in the DCM literature and amount to a few references in total. Some of the disparity in quantity of publications per disorder is likely due to the relative prevalence of certain disorders, perceived public health need and thus historical availability of funding. Relative to the other types of disorders and considering prevalence, substance use disorder appear to be particularly under-represented among DCM results – not to mention also with zero reports of self-connectivity findings. In all likelihood, the representation of substance use disorders in this list will increase most notably in the future given renewed recent interest, awareness of prevalence and increasing public health concern, as well as associated increase in research funding available.

Perhaps most noteworthy is the total absence of reported self-connectivity findings between the years of 2007–2011. Among subsequent years, only a minority of references included mention (25.9%). Even fewer articles include actual discussion (23.4%) or a graphical representation of self-connectivity findings (17.3%). So, even when results are being reported in the literature, they may not be receiving full consideration among other interregional results that have historically received greater focus. Overall, the historical trend points to a somewhat recently emergent tendency of reporting these results in some form, beginning in 2012 and continuing more later in the 2010s. Even in 2015 – the year with the highest incidence of self-connectivity findings being reported – only 5 of the 13 references reviewed (38.4%) for that year included mention of self-connectivity findings. This number was lower in 2016 (2 of 8, 25%) but the overall trend appears to demonstrate a non-linear but increasing adoption for the time being, perhaps due to ongoing efforts of experts in the field in providing additional guidance on interpreting and reporting such findings as well as increasing awareness over time.

The lack of statistically significant differences in the above study characteristics (e.g., number of subjects, ROI, number of extrinsic connections, etc.) may not be altogether surprising given that the DCM methodology may be otherwise consistently applied across the time period specified. Expectations for appropriate powering of neuroimaging studies have increased with time but such expectations are likely sustained at a high level through the specified interval. A tendency to report full models (perhaps with the historically apparent exception of self-connectivity findings) would indicate a similar number of ROIs and extrinsic connections. The later versions of SPM cited for studies reporting self-connectivity findings is consistent with the relative recency of consistent reporting of these results.

We acknowledge that other neuroimaging modalities (EEG and MEG) may have more robust inclusion and discussion of self-connectivity findings. Indeed, our literature search revealed a number of such references which were ultimately excluded in favor of focusing on fMRI findings for this mini-review. Perhaps this topic could be expanded further in a subsequent technical review or interval update which would include discussion of the factors which may result in differences in reporting self-connectivity findings across different imaging modalities.

## Conclusion

Despite a sufficient technical literature on the topic, there appears to be a tendency in the neuropsychiatric DCM literature to under-report self-connectivity findings. A number of possible explanations may arise for this observation including confusion about interpreting this kind of result in the context of perhaps “more interesting” inter-regional connections; only more recently emerging guidance on interpretation of self-connectivity findings; as well as a historically greater interest in “mapping out circuits” across the brain versus parsing out the intra-regional and inter-regional components in those circuits. Our review of the literature and consideration of our own self-connectivity findings suggest that self-connections represent an integral but under-reported part of DCM analysis. We encourage neuropsychiatric researchers – and particularly substance use disorder researchers – to reconsider the role of self-connectivity findings in future analyses.

## Author Contributions

AS, LM, and JS developed the literature review criteria. AS conducted the literature review and analysis. AS, LM, JS, KW, and FM wrote the manuscript. All authors contributed to the article and approved the submitted version.

## Conflict of Interest

The authors declare that the research was conducted in the absence of any commercial or financial relationships that could be construed as a potential conflict of interest.

## Publisher’s Note

All claims expressed in this article are solely those of the authors and do not necessarily represent those of their affiliated organizations, or those of the publisher, the editors and the reviewers. Any product that may be evaluated in this article, or claim that may be made by its manufacturer, is not guaranteed or endorsed by the publisher.
